# Lead Colic

**Published:** 1879-01

**Authors:** 


### LEAD COLIC

Is a disease to which painters, and workers in red and white lead, are subject, causing severe pains, tedious sickness, and often death. The disease is partially owing perhaps to breathing the fumes, but mainly from particles taken into the stomach by the food which is handled. Workmen can effect a total exemption from the disease by attending rigidly to three things.

*First.* Keep the finger nails trimmed closely so as to prevent particles of lead from collecting under them and transference to the bread in eating it.

*Second.* Wash the hands well with soap and water, and rinse the mouth before eating.

*Third.* Drink half a pint of sweet milk at each meal to antagonize the influence of any particles of lead which may find their way into the stomach. It has been found in thousands of cases that an habitual attention to these things secures an entire exemption from lead colic.

---

Attention to the Aged. — Nothing more distinctively marks the gentlemen or lady than deference to gray hairs, whatever may be the garb. It is a beautiful sight to see a boy promptly resign his seat to an old man; nothing sweeter than for a little girl or a young lady to be swift to assist an old woman. If it could be known how the old appreciate these things, how it cheers their hearts and lifts them up with thankfulness, the young would emulate one another in embracing any opportunity offered to be first to proffer a courtesy. And a thousand times swifter would all of us be to show our alacrity in this direction.

A few years ago one of the most accomplished young ladies of New York was a passenger on a steamer, returning from a European tour, her position in society was well known on board and she had many admirers who were very assiduous in their attentions to her, but notwithstanding all these she was noticed by a bank president never to miss an opportunity to show her deference towards the older passengers, and to put herself to considerable inconvenience to give assistance to those who needed it. "That young lady will make a good wife for my son," thought the banker to himself. He brought about an introduction before they reached New York and in due time they were married. The fashionable young lady to a millionaire's son, himself a partner in one of the oldest firms in the city. And all because this young lady had a heart for civilities to old people. That old man! what disappointments he has encountered in his long journey; what sorrows felt; what agonies endured; how many loved ones he has covered up in the grave! And that old woman, husband dead, children all buried or far away, life's flowers all faded, the friends of her youth all in their graves, and she waiting to go too, ought we to ever miss an opportunity of showing them an attention, of proffering a kindness or lighting up a smile by a courteous deed?

